# The Circulatory Risk in Communities Study (CIRCS): A Long-Term Epidemiological Study for Lifestyle-Related Disease Among Japanese Men and Women Living in Communities

**DOI:** 10.2188/jea.JE20180196

**Published:** 2019-03-05

**Authors:** Kazumasa Yamagishi, Isao Muraki, Yasuhiko Kubota, Mina Hayama-Terada, Hironori Imano, Renzhe Cui, Mitsumasa Umesawa, Yuji Shimizu, Tomoko Sankai, Takeo Okada, Shinichi Sato, Akihiko Kitamura, Masahiko Kiyama, Hiroyasu Iso

**Affiliations:** 1 Department of Public Health Medicine, Faculty of Medicine, and Health Services Research and Development Center, University of Tsukuba, Tsukuba, Ibaraki, Japan; 2 Osaka Center for Cancer and Cardiovascular Disease Prevention, Osaka, Japan; 3 Public Health, Department of Social Medicine, Osaka University Graduate School of Medicine, Suita, Osaka, Japan; 4 Yao Public Health Center, Yao City Office, Yao, Osaka, Japan; 5 Department of Public Health, Dokkyo Medical University, Mibu, Tochigi, Japan; 6 Department of Public Health and Nursing, Faculty of Medicine, University of Tsukuba, Tsukuba, Ibaraki, Japan; 7 Chiba Prefectural Institute of Public Health, Chiba, Japan; 8 Research Team for Social Participation and Community Health, Tokyo Metropolitan Institute of Gerontology, Tokyo, Japan

**Keywords:** community intervention, coronary heart disease, follow-up study, stroke

## Abstract

The Circulatory Risk in Communities Study (CIRCS) is an ongoing community-based epidemiological study of lifestyle-related disease involving dynamic prospective cohorts of approximately 12,000 adults from five communities of Japan: Ikawa, Ishizawa and Kita-Utetsu (Akita Prefecture), Minami-Takayasu (Osaka Prefecture), Noichi (Kochi Prefecture), and Kyowa (Ibaraki Prefecture). One of the most notable features of CIRCS is that it is not only an observational cohort study to identify risk factors for cardiovascular diseases (CVD), such as stroke, coronary heart disease, and sudden cardiac death, but it also involves prevention programs for CVD. Using basic, clinical, epidemiological, and statistical techniques, CIRCS has clarified characteristics of CVD and the related risk factors to develop specific methodologies towards CVD prevention in Japanese middle-aged or older adults for more than half a century.

## ORIGIN OF THE COHORT

**Figure 1.**
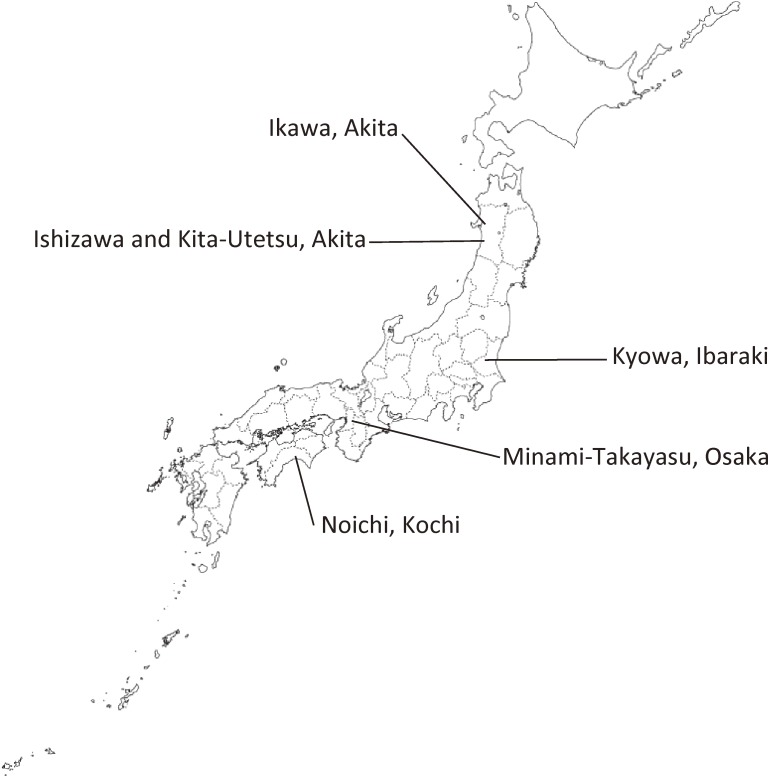
Location of study fields of the CIRCS

The Circulatory Risk in Communities Study (CIRCS) is an ongoing community-based epidemiological study using dynamic prospective cohorts involving approximately 12,000 persons per 4-to-6-year period (more than 65,000 people in total) in five Japanese communities: (1) Ikawa in Akita Prefecture (northeastern rural community); (2) Ishizawa and Kita-Utetsu, Honjo (presently Yuri-Honjo) City in Akita Prefecture (northeastern rural communities); (3) Minami-Takayasu, Yao City in Osaka Prefecture (mid-western suburban community); (4) Noichi, Konan City in Kochi Prefecture (western rural community); and (5) Kyowa, Chikusei City in Ibaraki Prefecture (mid-eastern rural community) (Figure 1).

In 1963, Dr. Yoshio Komachi, a physician and epidemiologist of the Osaka Medical Center for Cancer and Cardiovascular Diseases (presently Professor Emeritus of the University of Tsukuba), launched demonstrative surveys to reveal which lifestyle activities, especially those related to traditional Japanese lifestyle, were adversely associated with stroke, the leading cause of death in Japan in the 1960s, and also how to prevent it.^1^ Komachi and his colleagues started a comparative survey between communities in Akita and Osaka, where, nationally, mortality from stroke had been the highest and lowest, respectively. In 1969, they added the community of Noichi and, in 1981, they added the community of Kyowa as new survey fields. The surveys have been conducted annually, and the survey methods have been unified across those communities. The annual surveys and follow-up work in Ishizawa/Kita-Utetsu and Noichi terminated in 1987 and 2005, respectively. Community registration of stroke and coronary heart disease and mortality surveillance has been done for more than 55 years. Since 1999, registration was started for disabling dementia, based on the Japanese Long Term Care Insurance system.

In the initial ecological and cross-sectional studies by Komachi and his colleagues, which compared the incidence of stroke and coronary heart disease between rural Akita and suburban Osaka, they found that the incidence of stroke (especially that of cerebral hemorrhage), blood pressure levels, and salt intake were much higher in Akita, while the incidence of coronary heart disease, serum total cholesterol levels, and fat and protein intakes were lower. They observed that the lifestyle of people in Akita in the 1960s and 70s was characterized by a high consumption of traditional Japanese foods, such as a large intake of rice, miso soup, salt-preserved fish, pickles, soy sauce, and salt, and a small intake of animal products, as well as heavy agricultural work. Among traditional Akita farmers, lean middle-aged men were more likely to suffer from stroke. Based on their observations, Komachi and his colleagues hypothesized that the most important determinant of stroke was hypertension and not hyperlipidemia or diabetes in Japanese. They also hypothesized that the association between serum total cholesterol levels and cardiovascular disease (CVD) may not be linear, and that very low cholesterol levels, as well as very high blood pressure levels, would carry an adverse impact on risk of stroke, especially intraparenchymal hemorrhage. They also found that high cholesterol levels are positively associated with risk of coronary heart disease, confirming the findings of previous frontier studies: the Minnesota Business and Professional Men’s,^2^ Framingham Heart,^3^ and Seven Countries studies.^4^

Komachi’s U-shaped hypothesis caused strong debate, coming under criticism from some western researchers who suggested that the Japanese physicians may have misdiagnosed coronary heart disease as stroke. They also questioned the accuracy of measurement of the serum total cholesterol. In response, Komachi and his colleagues, as well as the Hisayama Study group, demonstrated that stroke diagnosis was validated using autopsy findings.^5^^,^^6^ They standardized the epidemiological criteria of stroke and coronary heart disease according to the World Health Organization (WHO) criteria^7^ since the 1960s, and have used the findings of computed tomography (CT)/magnetic resonance imaging (MRI) for the detailed classification of stroke types since the 1980s.^8^^,^^9^ In 1975, their lipid laboratory participated in the CDC-NHLBI Lipid Standardization Program of the United States’ Centers for Disease Control and Prevention (CDC) and National Heart, Lung, and Blood Institute (NHLBI), and since then the laboratory has become well-known for its accuracy and precision.^10^ From 1992, the laboratory became a member of the Cholesterol Reference Method Laboratory Network (CDC/CRMLN).

The U-shape hypothesis gained wider acceptance after 1989 through the publication of a prospective study conducted in Akita as a part of CIRCS,^11^ and a cohort study, the Multiple Risk Factor Intervention Trial (MRFIT), in the United States,^12^ which independently showed the inverse association between low total cholesterol levels and risk of intraparenchymal hemorrhage. Following these reports, the NHLBI held an international conference on low blood cholesterol in 1990 to review and discuss existing data on the U-shaped relationship between total cholesterol levels and mortality from CVD and other causes from 19 cohort studies worldwide.^13^ The inverse association between the total or low-density lipoprotein (LDL) cholesterol levels and risk of intraparenchymal hemorrhage has since been repeatedly replicated in meta-analyses and cohort studies.^14^

The study name ‘CIRCS’ was first used in 2008, before which there was no specific study name; sometimes it was referred to as the ‘Akita-Osaka Study’^15^ or ‘Osaka Medical Center Study’.^16^ To search the PubMed database for CIRCS papers published on or before 2008, it is useful to use the phrase: “Shimamoto T and Tsukuba”. The key features of CIRCS have previously been introduced in the literature.^17^^,^^18^

CIRCS has been conducted as a part of community prevention program and has, therefore, been sponsored mainly by each local municipality. Osaka Prefecture, the Ministry of Education, Culture, Sports, Science and Technology, the Ministry of Health, Labour, and Welfare, and other relevant organizations have also supported CIRCS.

## SCOPE AND FEATURES

CIRCS is one of the oldest epidemiological studies of CVDs in Japan. When CIRCS was first started in the 1960s, strategies for prevention of CVDs, especially of stroke, were not yet established in Japan. The public health mission of CIRCS was to find out ways to improve stroke prevention in community settings. A systematic mass screening was begun, which constituted measurements of blood pressure, electrocardiogram, ocular funduscopy, and blood test, all of which were not commonly carried out at that time, and the technique for physical examination of the general population had also not yet been established. CIRCS strictly standardized each test as an exposure factor according to international standards. Participants were interviewed about lifestyle-related factors, such as employment, smoking, alcohol, diet, and physical activity. The main outcome measures were incidence of stroke (intraparenchymal hemorrhage, subarachnoid hemorrhage, and cerebral infarction), coronary heart disease (myocardial infarction, effort angina pectoris, and sudden cardiac death), and dementia. Based on CT/MRI imaging, cerebral infarction was further classified in subtypes (lacunar, large-artery occlusive and embolic infarctions). CIRCS is composed of several community-based cohorts with different lifestyles and living environments, which makes it possible to compare incidence of CVD and its risk factors to identify different or common features among the participating communities and to clarify the characteristics of CVD in Japan.

CIRCS has a number of other important characteristics. First, as well as the hard endpoints mentioned above, through its annual surveys CIRCS has been used to identify intermediate outcomes, including hypertension, diabetes mellitus, atrial fibrillation, chronic kidney disease, and gout. Second, repeat measures or trajectory analyses can be done based on annual survey data. Third, dietary information, based on 24-hour dietary recall, is available. Fourth, data from over more than 5 decades can be analyzed. Finally, serum samples dating back from the 1980s have been stored and are available for research.

### Building and maintaining the cohort

The first survey was conducted in 1963 and included the following examinations: medical history, height, weight, blood pressure, physical examination, urinalysis, laboratory measurements of serum total cholesterol and protein, electrocardiogram, color photographs of the right ocular fundus, and an interview for assessing lifestyle factors.^11^ This examination system was novel in Japan at that time. These ongoing surveys, alongside some additional examinations, have been conducted annually with the aim of finding of novel risk factors and measures for prevention of CVD.

There follows a brief description for each of the major examinations. (1) Trained physicians, nurses, or technicians measured systolic and fifth-phase diastolic blood pressures using a standard mercury sphygmomanometer placed on the right arm of the participants who had been resting for at least 5 minutes prior to the measurement. (2) Blood samples were collected while the participants were quietly seated. The blood was drawn into siliconized vacuum tubes and was generally kept standing for 10 to 30 minutes at room temperature to let the blood clot before being centrifuged at 1,300 to 1,500 × g for approximately 15 minutes. The serum was separated and stored at −80°C awaiting laboratory analyses. As examples of the laboratory analyses performed, serum total cholesterol was measured using the Zac-Henly method from 1963 to 1974, the Liebermann-Burchard direct method from 1974 to 1986, and the enzymatic method from 1986 to the present. Glucose was measured using the cupric-neocuproine method from 1975 to 1986, the hexokinase method from 1986 to 1993, the glucokinase method from 1993 to 2001 period, and the hexokinase method from 2001 to the present. All blood samples were measured using standardized protocols at the laboratory of the Osaka Medical Center for Health Science and Promotion (currently, the Osaka Center for Cancer and Cardiovascular Disease Prevention); and this consistence over a 55-year period has allowed for the investigation of long-term trends. (3) Electrocardiogram was measured in the supine position. Two physicians independently coded each record of electrocardiogram, based on the Minnesota Code. If the codes were in agreement, they were accepted, while disputed codes were discussed by the two physicians, and if necessary, a third experienced physician arbitrated. (4) Color photographs taken of the right ocular fundus were independently read and coded by two trained physicians using a modified Scheie classification. Agreement and disagreement over classifications were dealt with in the same manner as described for electrocardiograms.

With regard to stroke and coronary heart disease registration, CIRCS investigated the following ascertainment sources in addition to all hospitalized cases: baseline surveys, death certificates, ambulance records, national insurance claims, and reports from local physicians, public health nurses, and health volunteers. In order to validate the diagnoses, all living patients were either visited or invited to baseline surveys or their medical history was obtained from their families. Trained physicians reviewed medical records in the local clinics and hospitals. In the cases of deceased participants, their histories were obtained from their families and/or attending physicians, and medical records were reviewed. Modified criteria of the National Survey of Stroke^7^ and the WHO Expert Committee^9^ were used for the diagnoses of coronary heart disease and stroke, respectively. The CT and/or MRI images were available in approximately 60% of stroke cases in the 1980s and over 90% thereafter, and these images were used to classify the cases into subtypes based on the affected site.^8^

### First publication

The first CIRCS-related paper was published in 1964 reporting the findings of health examination in two areas of Akita prefecture (Ikawa and Yuri) and two areas of Osaka prefecture (Akegawa area in Yao and Nose).^19^ At that time, moderate-to-severe hypertension was more prevalent in Akita than in Osaka: the prevalence of hypertension defined using WHO criteria (systolic blood pressure ≥160 mm Hg or diastolic blood pressure ≥95 mm Hg) was around 25% in Akita and around 10% in Osaka among residents aged 40–54 years. Among those aged 55–64 years, the corresponding prevalence was around 50% in Akita and around 30% in Osaka. Moreover, in both areas, half of moderate-to-severe hypertension was categorized into severe hypertension (systolic blood pressure ≥180 mm Hg or diastolic blood pressure ≥110 mm Hg). Such a large difference in blood pressure levels between Akita and Osaka suggested a large regional difference in annual incidence rate of stroke, which was indeed reported several years later.^20^ The annual incidence rate of stroke was about 2-fold higher in Akita than that in Osaka: among residents aged 40–69 years, the annual incidence rate was 5.0 cases/1,000 population in Nose (Osaka), 5.6 cases/1,000 population in Yao (Osaka), 10.4 cases/1,000 population in Ikawa (Akita), and 12.3 cases/1,000 population in Yuri (Akita). This regional inequality regarding stroke incidence justified the need for intensive intervention programs for stroke, especially in Akita.

### Most cited papers

**Figure 2.**
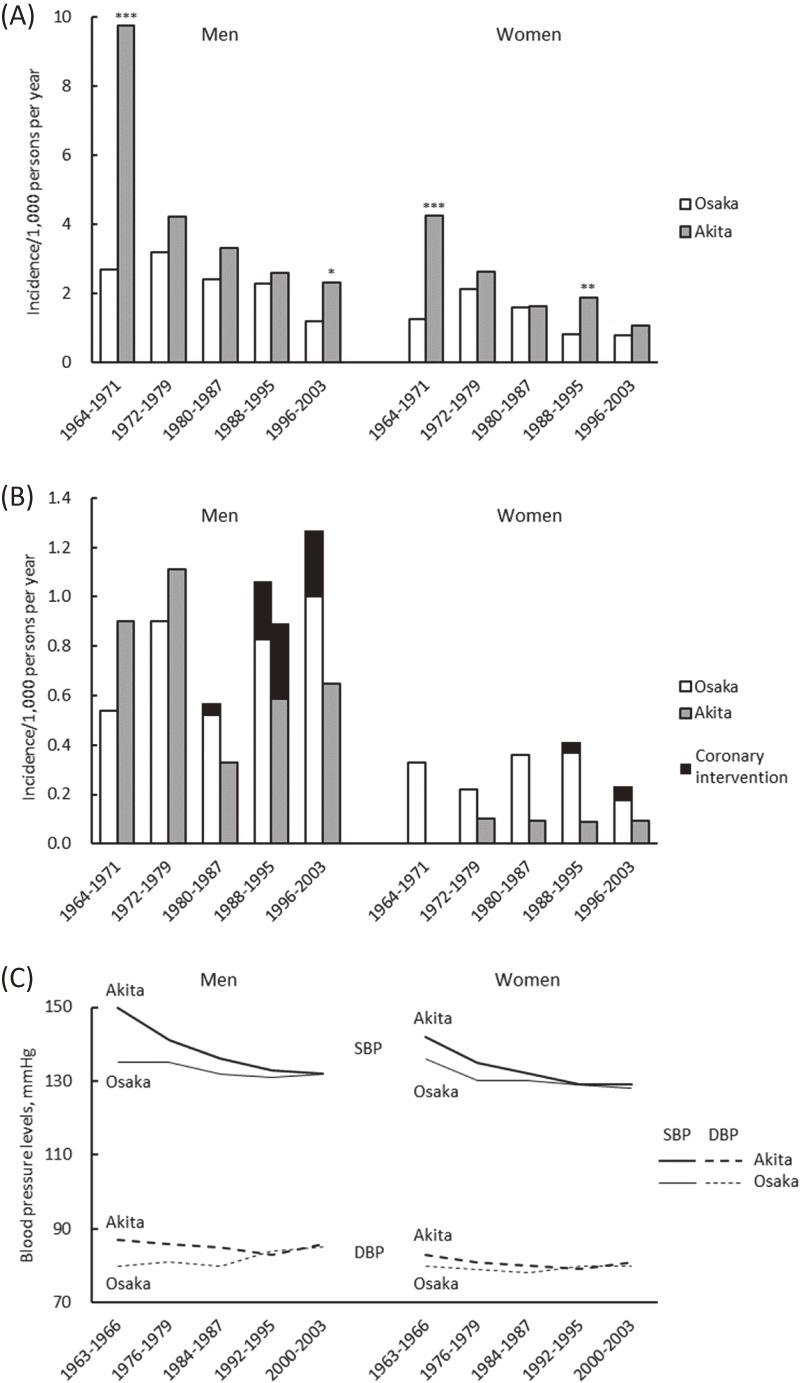
Trends for age-adjusted incidence of stroke (A), coronary heart disease (B) and blood pressure levels (C). ^*^P < 0.05, ^**^P < 0.01, and ^***^P < 0.001 for differences between Akita and Osaka. (Modified from Kitamura, et al. *J Am Coll Cardiol* 2008;52:71–79)

Two decades after the establishment of CIRCS, the time trend of CVD incidence and its risk factors in Ikawa, a rural town of Akita prefecture between 1964–68 and 1979–83 was reported by Shimamoto et al,^11^ which is the most cited paper from CIRCS. That study was updated up to 2003 and expanded to include a district of Yao City, Osaka Prefecture, a suburban area of mid-western Japan.^15^ In both Ikawa and Yao, the age-adjusted annual incidence of total stroke (cases/1,000 population) among both men and women aged 40–69 years was declining through 2003: these figures changed from 9.7 for men and 4.2 for women in 1964–1971 to 2.3 for men and 1.1 for women in 1996–2003 in Ikawa; and from 2.7 for men and 1.3 for women to 1.2 for men and 0.8 for women in Yao, respectively (Figure 2A). Regarding coronary heart disease, the age-adjusted incidence in Ikawa decreased slightly through 2003, but the trend was not statistically significant due to the low incidence (Figure 2B). In contrast to Ikawa, the incidence of coronary heart disease in Yao significantly increased among men (from 0.6 cases/1,000 population in 1980–1987 to 1.3 cases/1,000 population in 1996–2003). In terms of traditional cardiovascular risk factors, systolic and diastolic blood pressure levels (Figure 2C) and the proportion of patients with hypertension decreased among men and women in both areas, while mean levels of serum total cholesterol increased. The proportion of patients with medication use for hypertension and high cholesterol also increased. Among men, the prevalence of smoking and heavy drinking declined. During the early period in Ikawa, serum levels of total protein was elevated, which was in part reflected by an increased daily meat intake from 5.8% of energy to 7.1% of energy among men aged 40–59 years.^11^

### Other features

One of the most notable features of CIRCS is, again, not only its usefulness for identifying risk factors for CVD, but also as a platform for the introduction of prevention programs for CVD and their risk factors using basic, clinical, epidemiological, and statistical techniques.

**Figure 3.**
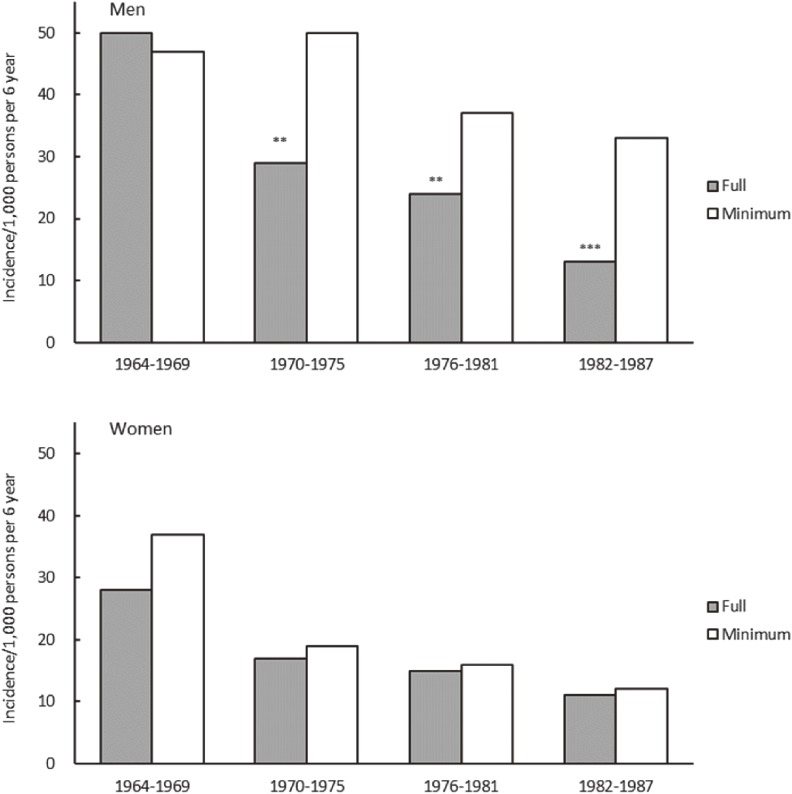
Trends for age-adjusted incidence of stroke in full and minimal intervention communities. Difference from the minimal intervention community: ^**^P < 0.01, ^***^P < 0.001. (Data from Iso, et al. *Stroke* 1998;29:1510–1518)

**Figure 4.**
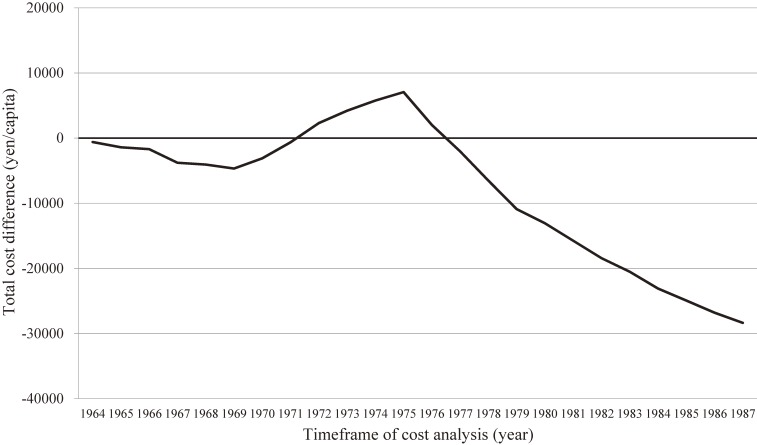
Cost analyses of the hypertension detection and control program, 1964–1987. X-axis: Time frame of cost analysis (*t*). Y-axis: Total cost difference by year defined as follows: Total cost difference = $\sum_{year=1964}^{t}\left\{C_{A}{\left(1-0.04\right)}^{year-1964}-C_{B}{\left(1-0.04\right)}^{year-1964}\right\}$, *t* = 1964–1987, where *C_A_* stands for total cost (after adjustment for consumer price index) in the full intervention community and *C_B_* stands for that in the minimal intervention community. Discount rate was 4% per year. (Reprinted from Yamagishi, et al. *J Hypertens* 2012;30:1874–1879)

Because CIRCS is a dynamic cohort study, which has consistently performed baseline surveys and has conducted CVD surveillance every year since 1963, it has also allowed for the reporting of trends for stroke and coronary heart disease incidences and their risk factors^11^^,^^15^^,^^21^ and impacts of health education programs on hypertension^22^ and hypercholesterolemia.^23^ There follows an introduction to two examples of prevention programs that grew out of CIRCS. First, in a report of the effects of a long-term hypertension control program for stroke prevention in communities^24^ (Figure 3) that compared two communities for trends in blood pressure levels and stroke incidence and prevalence between 1963 and 1987, Ikawa, one of two communities, received a full range of community-wide hypertension interventions, while the other had a minimal intervention. In men, stroke incidence and prevalence declined in the full-intervention area (Ikawa) more than in the minimal-intervention community, and differential trends in systolic blood pressure levels appeared to explain the larger decline in stroke. Second, in a report on the cost-effectiveness of this long-term hypertension control program^25^ (Figure 4) costs of public health services and of treatment for patients with hypertension or stroke in the full-intervention community (Ikawa) and minimal-intervention communities were compared. It was found that the program in the full-intervention community became cost saving 13 years after its initiation; the incremental costs reduced by 28,358 Japanese yen per capita over 24 years.

CIRCS has led to the identification of several novel risk/preventive factors for CVD: lipids (eg, serum fatty acids composition^26^^,^^27^ and high-density lipoprotein (HDL)-cholesterol particle size^28^), glucose tolerance (non-fasting blood glucose^29^^,^^30^), other biochemical factors (serum liver/biliary tract enzymes,^31^^,^^32^ serum homocysteine,^33^ serum C-reactive protein,^34^ and adiponectins^35^), hematological factors (leukocyte counts^36^), fibrinolytic factors (plasma fibrinogen^37^^–^^39^), electrocardiographic factors (ischemic abnormalities^40^^,^^41^ and Brugada-type electrocardiogram^42^), other physiological factors (carotid atherosclerosis^43^ and ankle-brachial blood pressure index^44^), dietary factors (fat and protein intakes^45^), psychosomatic factors (depressive symptoms^46^), height,^47^ snoring,^48^ metabolic syndrome,^49^^,^^50^ chronic kidney disease,^51^ and subclinical end-organ damage,^52^ as well as traditional risk factors (eg, alcohol,^53^^–^^55^ smoking,^56^ blood glucose/diabetes,^57^^,^^58^ blood pressure,^1^^,^^5^^,^^11^^,^^59^ total-,^1^^,^^5^^,^^11^ LDL-,^60^ non-HDL-^61^ and HDL-cholesterols,^62^^,^^63^ and triglycerides^64^^,^^65^). Recent reports included risk or preventive factors for dementia, such as smoking,^66^ C-reactive protein,^67^ serum coenzyme Q10,^68^ serum α-linoleic acid,^69^ and retinal vascular changes.^70^ Cross-cultural comparison studies of lipids,^71^^–^^73^ hemostatic factors,^74^^–^^77^ serum sialic acid,^78^ and sleep-disordered breathing^79^ with American populations have also been conducted. CIRCS has also been involved in several international or domestic collaborative studies, such as the Prospective Studies Collaboration,^80^ Fibrinogen Studies Collaboration,^80^ Emerging Risk Factors Collaboration,^81^ Chronic Kidney Disease Prognosis Consortium,^82^ Japan Arteriosclerosis Longitudinal Study,^83^ Japan Arteriosclerosis Longitudinal Study-Existing Cohorts Combine,^84^ and Evidence for Cardiovascular Prevention from Observational Cohorts in Japan Study.^85^ These studies have contributed to building evidence on prevention of CVD not only for Japanese, but also for people across the world.

### Historical impact on global and local health

During the past half century, CIRCS has continued to provide scientific evidence on issues of public health in Japan. One of the important findings that CIRCS showed is that the fact stroke is preventable via screening and controlling hypertension as well as through lifestyle modifications, such as reduction of salt intake, improvements of nutritional balance, and proper rest and physical activity. Based on the CIRCS findings, the Japanese government launched the first stroke prevention program across a few model municipalities in 1969, which was mainly composed of hypertension screening.^86^ Since then, the program was gradually extended to other municipalities and eventually the cardiovascular screening system was established in 1982 under the Health and Medical Service Act for the Aged, which made every municipality responsible for offering screening and preventive activities.^86^ Since 2008, a screening system by health insurers has been conducted, which focuses on prevention and control of metabolic syndrome under the Act on Assurance of Medical Care for Elderly People.

The findings from CIRCS, an ongoing dynamic cohort study, can be applicable not only to the other East Asian countries, but also to Southeast Asian and African countries with high stroke and low coronary heart disease. The CIRCS measures were introduced by the WHO in 2013 as a model program to defeat stroke.^87^

This seminal epidemiological study revealed that risk factors and characteristics of CVD were in part different between Japan and the United States/Europe and that it was necessary to develop distinct ways of prevention for CVD in the Japanese context. CIRCS has provided scientific evidence to aid the development of national health projects including the basic health checkup systems aimed at prevention of CVD and promotion of health in communities.

### The CIRCS Investigators

CIRCS has been led by Yoshio Komachi (Professor Emeritus of University of Tsukuba), Minoru Iida, Hideki Ozawa, the late Takashi Shimamoto (Professor Emeritus of University of Tsukuba), the late Masamitsu Konishi (Professor Emeritus of Ehime University), and Yoshinori Ishikawa. The present investigators of CIRCS are as follows: Hiroyasu Iso, Hironori Imano, Renzhe Cui, Isao Muraki, Hiroyuki Noda, and Hiroshige Jinnnouchi (Osaka University), Masahiko Kiyama, Takeo Okada, Yuji Shimizu, Mina Hayama-Terada, and Yasuhiko Kubota (Osaka Center for Cancer and Cardiovascular Disease Prevention), Tomoko Sankai, Kazumasa Yamagishi, and Mizuki Sata (University of Tsukuba), Isao Koyama and Masakazu Nakamura (National Cerebral and Cardiovascular Center), Isao Saito (Oita University), Koutatsu Maruyama (Ehime University), Shinichi Sato (Chiba Prefectural Institute of Public Health), Mitsumasa Umesawa and Masanori Nagao (Dokkyo Medical University), Takeshi Tanigawa and Ai Ikeda (Juntendo University), Tetsuya Ohira (Fukushima Medical University), and Akihiko Kitamura (Tokyo Metropolitan Geriatric Medical Center).
